# Transcriptomics Reveals the Inhibitory Effect of Scutellarin on PRRSV-Infected PAMs

**DOI:** 10.3390/v17111460

**Published:** 2025-10-31

**Authors:** Guidong Zhang, Teng Tu, Yanwei Li, Yueyan Zeng, Mingpeng Hu, Chengchao Du, Zexiao Yang, Xueping Yao, Dishi Chen, Tian Shi, Yin Wang

**Affiliations:** 1 College of Veterinary Medicine, Sichuan Agricultural University, Chengdu 611130, China; 2023203024@stu.sicau.edu.cn (G.Z.); 2022203032@stu.sicau.edu.cn (Y.L.); zengyueyanstone@gmail.com (Y.Z.); 18728159022@163.com (M.H.); 18782680392@163.com (C.D.); 13643@sicau.edu.cn (Z.Y.); 13577@sicau.edu.cn (X.Y.); 2 Division of Student Affairs, Chengdu University of Technology, Chengdu 610059, China; 2019303092@stu.sicau.edu.cn; 3 Sichuan Provincial Animal Disease Prevention and Control Center, Chengdu 610047, China; cds815@sina.com; 4 Chengdu Animal Disease Prevention and Control Center, Chengdu 610041, China; stflee@126.com

**Keywords:** porcine reproductive and respiratory syndrome virus, scutellarin, transcriptomic analysis

## Abstract

Porcine reproductive and respiratory syndrome (PRRS) is a highly contagious epidemic caused by the porcine reproductive and respiratory syndrome virus (PRRSV). Characterized by reproductive disorders in pregnant sows and respiratory symptoms in pigs of all ages, it poses a severe threat to the global swine industry. In recent years, the high mutation rate of PRRSV has increasingly limited the effectiveness of vaccines against it, prompting the search for new anti-PRRSV drugs. scutellarin (SCU), a natural flavonoid compound extracted from the medicinal plant *Scutellaria baicalensis*, possesses multiple biological activities. Its antiviral effects have been demonstrated in numerous studies; however, its inhibitory activity against PRRSV and the underlying mechanism remain unknown. In this study, through in vitro cell experiments, we found that scutellarin significantly inhibits PRRSV infection in PAMs. Furthermore, it directly acts on PRRSV to exert antiviral effects. Transcriptomic analysis suggests that scutellarin may exert its anti-PRRSV effects by regulating host immunity and anti-inflammation through immune-related signaling pathways, including the complement and coagulation cascades, ECM-receptor interaction, Rap1 signaling pathway, and PI3K-Akt signaling pathway.

## 1. Introduction

Porcine Reproductive and Respiratory Syndrome (PRRS, also known as blue ear disease) is a highly contagious disease caused by the Porcine Reproductive and Respiratory Syndrome Virus (PRRSV). It is characterized by reproductive disorders in pregnant sows and respiratory symptoms in pigs of all ages. In infected sows, the disease leads to reduced farrowing rates, with abortion, stillbirth, and weak piglet rates reaching 30% to 100%. In growing-finishing pigs, clinical signs include dyspnea, frequent secondary infections, slowed production, and reduced feed conversion efficiency [1,2,3]. The disease was first reported in the United States in 1987 and subsequently spread rapidly to the vast majority of pig-producing countries worldwide, severely jeopardizing the development of the global swine industry [4,5]. Porcine reproductive and respiratory syndrome virus (PRRSV) belongs to the family *Arteriviridae*, genus *Betaarterivirus*. The viral particles are spherical or ellipsoidal, enveloped, with a diameter of 45–80 nm. They contain an icosahedral-symmetric nucleocapsid structure. The genome is approximately 15.4 kb in length, consisting of a single-stranded, positive-sense, non-segmented RNA virus. The 5′ end features a cap-like leader sequence, and a poly(A) tail sequence at the 3′ end [6,7]. The PRRSV genome contains at least 10 overlapping open reading frames (ORFs) arranged from 5′ to 3′ as follows: ORF1a, ORF1b, ORF2a, ORF2b, ORF3, ORF4, ORF5a, and ORF5–ORF7. These ORFs encode at least 16 non-structural proteins (Nsp1α, Nsp1β, Nsp2–6, Nsp7α, Nsp7β, and Nsp8–12) and 8 structural proteins (GP2, GP3, GP4, GP5, M, N, and E proteins) [8,9,10,11]. In recent years, the high mutation rate of PRRSV viral proteins and the emergence of drug resistance to antiviral medications have increasingly limited our ability to control this disease. To address this issue, there is an urgent need for new antiviral drugs or compounds.

Scutellarin (SCU) is a natural flavonoid compound extracted from the medicinal plant *Scutellaria baicalensis*. It exhibits multiple pharmacological effects including anti-inflammatory [12,13,14], antibacterial [15,16], antitumor [17,18], antioxidant [19], and antiviral [20,21,22] effects. Numerous studies have demonstrated that scutellarin exhibits potent antiviral activity. Li Yuyun [21] discovered through in vitro experiments that scutellarin can specifically bind to phospholipid molecules on viral envelopes, inhibiting the fusion of viral envelopes with cell membranes, thereby exerting broad-spectrum antiviral activity against enveloped viruses. Coincidentally, Keum et al. [22] discovered during their screening of potential anti-SARS-CoV drugs that

Scutellarin could inhibit the SARS-CoV virus helicase, thereby interfering with viral replication. Although scutellarin has been proven to exhibit potent inhibitory effects against viruses such as SARS-CoV and HIV, its potential inhibitory activity against PRRSV and the underlying mechanism of action remain unexplored. Further research is required in this area.

In this study, we confirmed that scutellarin exhibits potent inhibitory activity against PRRSV. Furthermore, it directly interacts with PRRSV to exert its antiviral effects. Transcriptomic analysis revealed that scutellarin may modulate host immunity and inflammation by influencing immune-related signaling pathways, including the PI3K-AKT and Rap1 signaling pathways, thereby exerting its anti-PRRSV activity.

## 2. Materials and Methods

### 2.1. Cell Cultivation and Viral Infection

PAMs (PRRSV receptor CD163 and CD169 was expressed and transfected into primary alveolar macrophage passage cell line 3D4/21 (CRL-2843)) was maintained and provided by the Animal Quarantine Laboratory of Sichuan Agricultural University.

PAMs (expressing CD163 and CD169 receptors) were cultured in RPMI Medium 1640 supplemented with 15% fetal bovine serum (FBS), 100 U/mL penicillin, and 100 μg/mL streptomycin. The serum concentration in maintenance medium (MM) was reduced to 2%.

PRRSV SCSN2020 strain (GenBank accession number: OQ883907) belongs to the JXA1-Like strain. It was isolated and preserved by our laboratory and propagated in PAMs.

### 2.2. Establishment of the Quantitative Fluorescent PCR Standard Curve

#### 2.2.1. Design and Synthesis of Fluorescent Primers

Based on the nucleotide sequence of PRRSV SCSN2020, primers targeting a 609 bp amplification fragment were designed using SnapGene 8.0.1 software. These primers were synthesized by Beijing Qingke Biotechnology Co., Ltd., Beijing, China (Table A1 in Appendix A) for subsequent plasmid validation.

#### 2.2.2. Preparation and Identification of Positive-Strand Plasmids

RNA extraction and cDNA preparation were performed on the PRRSV SCSN2020 viral suspension using the Cofitt Universal Genomic RNA Kit (Chengdu Cofitt Biotechnology Co., Ltd., Chengdu, China). The target fragment was synthesized using the specific primers synthesized in Section 2.2.1 and recovered from the gel using a gel recovery kit. The recovered cDNA was used to construct a positive control plasmid using a homologous recombination kit. The resulting pcDNA3.1-M recombinant bacteria were cultured in expansion, and plasmid extraction was performed using a plasmid extraction kit. The concentration and purity of the obtained plasmid were measured using a NanoDrop2000(Thermo Fisher Scientific, Shanghai, China), and the copy number was calculated. Formula: (6.02 × 10^23^) × plasmid concentration (ng/μL) × 10^−9^/(pcDNA3.1-M gene length) × 660 = copies/μL.

#### 2.2.3. Measure and Plot the Standard Curve

The pcDNA3.1-M plasmid obtained in Section 2.2.2 was serially diluted from 1 × 10^−3^ copies/μL to 1 × 10^−10^ copies/μL, with three replicates per dilution gradient. The CT values were measured for each dilution, and a standard curve was plotted.

### 2.3. Determination of Cell Safety Concentration by Scutellarin

Dissolve scutellarin (PCS0329, Chengdu Zhibiao Huanchun Biotechnology Co., Ltd., Chengdu, China) in dimethyl sulfoxide (DMSO) is sterilized by filtration using a 0.22 μm filter tip. Dilute the sterilized scutellarin with cell culture medium (DMSO < 0.5%) to concentrations of 0.8 mg/mL, 0.6 mg/mL, 0.4 mg/mL, 0.2 mg/mL, 0.1 mg/mL, 0.05 mg/mL. Add to 96-well plates containing monolayer cells, with 4 replicates per concentration. Include a blank control and cell control group. Incubate at 37 °C for 48 h. Add CCK-8 solution to the 96-well plates, incubate for an additional hour, then measure the OD450. Calculate the drug inhibition rate. Drug inhibition rate (%) = (Cell control well OD value − Test well OD value)/(Cell control well OD value − Blank control well OD value) × 100%. Note: Cell control well (cells + medium + CCK-8 solution); Test well (cells + medium + CCK-8 solution + drug); Blank control well (medium + CCK-8 solution).

### 2.4. Mechanism of Scutellarin Action Against PRRSV

#### 2.4.1. Preventive Effect of Scutellarin Against PRRSV

After digesting PAMs with trypsin, uniformly plate them into a 24-well cell culture plate. Using cell culture medium, continue serial dilutions of the maximum safe concentration identified in the previous screening (2^0^, 2^−1^, 2^−2^, 2^−3^), 500 μL/well. Incubate at 37 °C for 4 h, then wash three times with PBS. Add SCSN2020 viral solution (MOI = 0.1), incubate for 1.5 h, then discard the viral solution. followed by three PBS washes. Add 500 μL maintenance medium and incubate at 37 °C in a 5% CO_2_ incubator for 24 h. For the control group, use maintenance medium instead. Extract total RNA from each group of cells and calculate viral copy numbers using q-PCR to determine the preventive effect.

#### 2.4.2. Determination of the Direct Effect of Scutellarin on PRRSV

After trypsin digestion, PAMs were uniformly seeded into 24-well cell culture plates. Viral suspension (SCSN2020, MOI = 0.1) was mixed with scutellarin diluted in a twofold serial dilution. The control group used maintenance medium instead. After incubation at 37 °C in a water bath for 1.5 h, the mixture was added to the 24-well plates (containing confluent cells). After 1.5 h incubation at 37 °C, the medium was discarded, cells were washed three times with PBS, and 500 μL maintenance medium was added for continued culture for 24 h. Total RNA was extracted from each group of cells. Viral copy numbers were calculated using q-PCR to determine the direct effect.

#### 2.4.3. Scutellarin Inhibition of PRRSV

After trypsin digestion, PAMs were uniformly seeded into 24-well cell culture plates. Viral suspension (MOI = 0.1) was added to the treatment wells, while the control group received maintenance medium instead. After incubation at 37 °C for 24 h, cells were washed three times with PBS. A 2-fold diluted scutellarin was added, and the plates were incubated for an additional 24 h in a 5% CO_2_ incubator. Total RNA was extracted from each group of cells. Viral copy numbers were quantified using q-PCR to determine the inhibitory effect.

#### 2.4.4. Scutellarin Adsorption of PRRSV

After digesting PAMs with trypsin, uniformly seed them into a 24-well cell culture plate. Mix the SCSN2020 viral suspension (MOI = 0.1) with serially diluted scutellarin in duplicate. For the control group, substitute with maintenance medium. Add the mixtures to the 24-well plate (containing confluent cells) and incubate at 4 °C for 1.5 h for adsorption. Discard the viral solution. and washed three times with PBS. Total RNA was extracted from each group of cells. Viral copy numbers were quantified using q-PCR to determine adsorption efficiency.

### 2.5. Transcriptomic Analysis of the Inhibitory Effect of Scutellarin on PRRSV

#### 2.5.1. Total RNA Extraction from Cell Samples and Library Quality Control

Sample preparation was performed using the method with the best efficacy described in Section 2.4. Groups included the treatment group (SCU + SCSN2020 virus solution + cells), the virus group (SCSN2020 virus solution + cells), and the control group (cells), each with 5 replicates. Total RNA was isolated using the TRIzol Total RNA Extraction Kit (TianGen Biochemical Technology Co., Ltd., Beijing, China), yielding > 2 μg of total RNA per sample. RNA quality was assessed via 0.8% agarose gel electrophoresis and spectrophotometry. High-quality RNA with a 260/280 absorbance ratio of 1.8–2.2 was used for library preparation and sequencing. Libraries were constructed by APExBIO following Illumina manufacturer protocols. In summary, polyA-tailed mRNA was captured using oligo(dT) magnetic beads. Captured mRNA was fragmented, reverse transcribed, and synthesized into double-stranded cDNA. The cDNA product was purified using the AMPure XP system (Beckman Coulter, Beverly, CA, USA). Following library preparation, enriched library fragments were amplified via PCR and selected based on fragment size (350–550 bp). Library quality was assessed using an Agilent 4200 Bioanalyzer (Agilent Technologies, Santa Clara, CA, USA). Libraries were sequenced on an Illumina Xplus platform (PE150) to generate raw data.

#### 2.5.2. RNA Data Analysis

Raw data were filtered using Fastp software (v0.23.2) to discard sequences containing adapters and low-quality bases. The resulting valid data were then aligned to the Suscrofa reference sequence using HISAT2, followed by transcriptome assembly and gene expression quantification with StringTie. Differentially expressed genes (DEGs) were identified using DEseq2 (for samples with biological replicates), with a screening threshold of |log2(FoldChange)| > 1 & padj < 0.05. ClusterProfiler performed functional enrichment analysis of significant DEGs for Gene Ontology (GO) and KEGG pathway categories, with features at *p* < 0.05 considered significant. Gene set enrichment analysis (GSEA) was performed using the clusterProfiler function within the package, with gene lists sorted by log2foldchange.

#### 2.5.3. RT-qPCR Validation

To validate the accuracy of transcriptome sequencing results, 21 differentially expressed genes were randomly selected for qRT-PCR analysis. β-actin served as the internal control gene. Primers were synthesized by Beijing Qingke Biotechnology Co., Ltd. (Appendix A).

### 2.6. Data Analysis

Experimental data were analyzed using GraphPad Prism 8.0.1 via two-way analysis of variance (ANOVA) or *t*-tests. *p* < 0.05, *, indicates significant difference; *p* < 0.01, **, indicates highly significant difference; *p* < 0.001, ***, indicates extremely significant difference; *p* > 0.05, ns, indicates no significant difference.

### 2.7. Analysis of Inflammation-Related Gene Expression Levels in PAMs Induced by PRRSV Following Treatment with Scutellarin

RNA was extracted from PAMs using a total RNA extraction kit. RT-qPCR was performed to detect mRNA levels of inflammation-related genes in each group, with β-actin as the internal control gene. Primers are listed in Appendix A.

## 3. Results

### 3.1. Establishment of the Quantitative Fluorescent PCR Standard Curve

The concentration of recombinant plasmid pcDNA3.1-M was determined using NanoDrop2000 to be 415 ng/μL. Based on the copy number formula, the copy number of the recombinant plasmid was calculated to be approximately 5.05 × 10^11^ copies/μL. Detection of the above 8 dilution levels (5.05 × 10^3^~5.05 × 10^10^ copies/μL) of quantitative standards showed good linearity between 5.05 × 10^3^ and 5.05 × 10^10^ copies/μL, with a correlation coefficient R^2^ of 0.9994. with the equation Ct = −3.574 × lg copies + 45.55.

### 3.2. Safety Concentration Determination of Scutellarin for PAMs

This study evaluated the inhibition rates of scutellarin against PAMs using concentrations of 800 μg/mL, 600 μg/mL, 400 μg/mL, 200 μg/mL, 100 μg/mL, and 50 μg/mL. As shown in Table 1, when the scutellarin concentration was 200 μg/mL, the inhibition rate of PAMs was less than 10%. Generally, we consider a cell inhibition rate below 10% to be non cytotoxic. Therefore, the maximum safe concentration of scutellarin for PAMs is 200 μg/mL.

### 3.3. Optimal Mode of Action of Scutellarin on PRRSV

#### 3.3.1. Preventive Effect of Scutellarin on PRRSV

**Figure 1:**
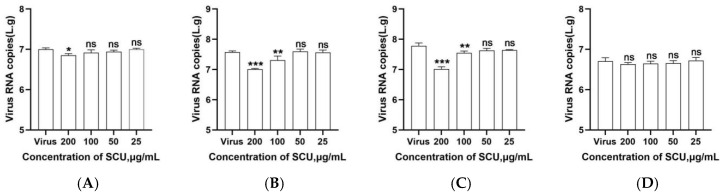
Results of scutellarin’s various modes of action against PRRSV. (**A**) Preventive effect; (**B**) Direct effect; (**C**) Inhibitory effect; (**D**) Adsorption effect. *p* < 0.05, *, significant difference; *p* < 0.01, **, *p* < 0.001, ***, highly significant difference; *p* > 0.05, ns, no significant difference.

To determine the optimal mode of action of scutellarin against PRRSV, this study employed serial dilutions based on the safe concentration of 200 μg/mL. The results are shown in Figure 1A. At a scutellarin concentration of 200 μg/mL, the viral copy number of the PRRSV SCSN2020 strain was significantly suppressed (*p* < 0.05), indicating that scutellarin may exert its anti-PRRSV effect by stimulating cells.

#### 3.3.2. Direct Effects of Scutellarin on PRRSV

When the PRRSV SCSN2020 strain viral suspension was mixed with scutellarin and incubated at 37 °C for 1.5 h, the results shown in Figure 1B indicate that at scutellarin concentrations of 100–200 μg/mL, the viral copy number of PRRSV was significantly reduced (*p* < 0.05). No significant differences were observed at other concentrations (*p* > 0.05). This indicates that scutellarin can directly interact with PRRSV to exert antiviral effects.

#### 3.3.3. Inhibitory Effect of Scutellarin on PRRSV

Scutellarin was added at equal concentrations to PAMs pretreated with PRRSV SCSN2020 viral solution. Results shown in Figure 1C indicate that scutellarin concentrations ranging from 100 to 200 μg/mL significantly reduced the viral copy number of PRRSV (*p* < 0.05), demonstrating scutellarin’s inhibitory effect on PRRSV infection in PAMs.

#### 3.3.4. Adsorption of PRRSV by Scutellarin

To determine the effect of scutellarin on PRRSV adsorption to PAMs, this study first incubated PRRSV with scutellarin at 4 °C for 1.5 h, followed by three washes with PBS. RT-qPCR analysis revealed that scutellarin did not reduce the viral copy number of PRRSV, as shown in Figure 1D.

### 3.4. Transcriptomics Analysis

#### 3.4.1. Gene Expression Analysis of Samples

**Figure 2:**
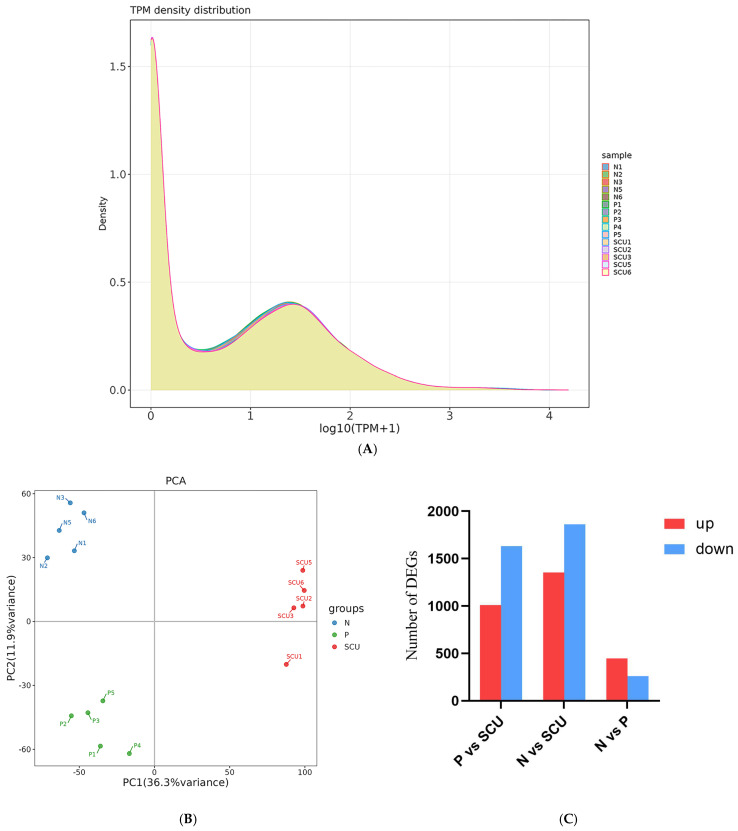

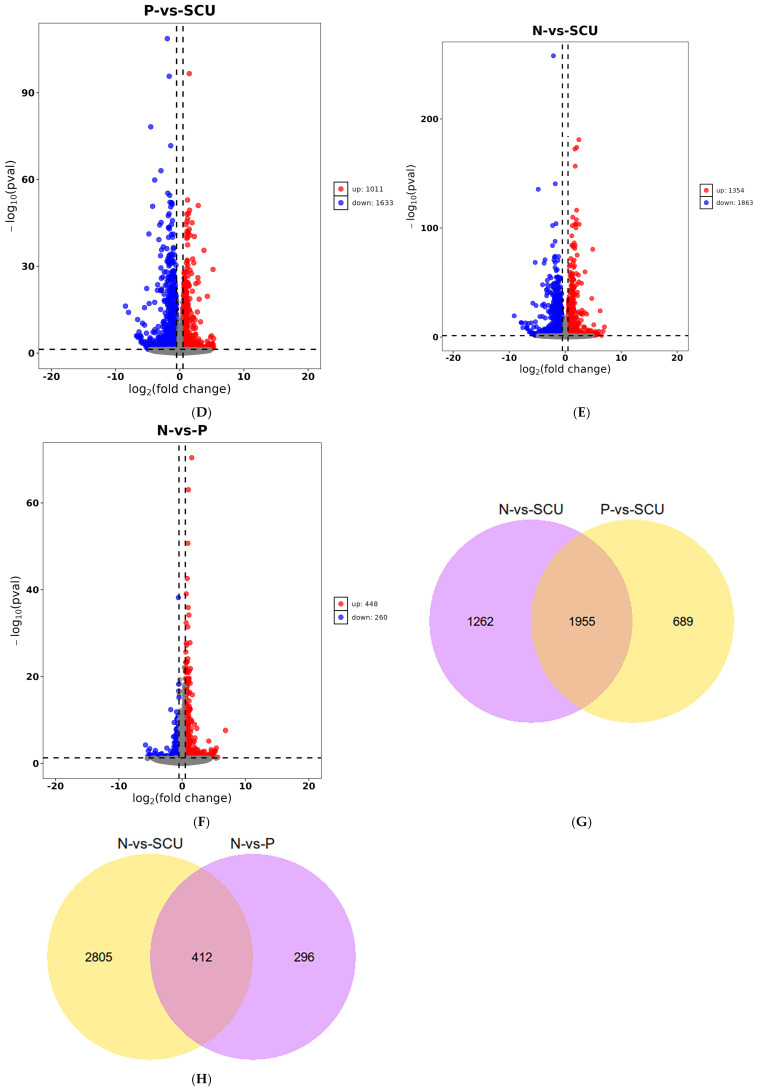
Differential analysis results. (**A**) Gene Expression Level Density Plot. Note:P vs. N: the control group vs. the virus group; P vs. SCU: the virus group vs. the treatment group; N vs. SCU: control group vs. the treatment group. (**B**) Principal Component Analysis Results Plot. (**C**) Number of differentially expressed genes (up- and down-regulated) between groups. (**D**–**F**) Volcano plots of differentially expressed genes. (**G**,**H**) Venn diagrams of genes with different expression patterns. Note: P vs. N: the control group vs. the virus group; P vs. SCU: the virus group vs. the treatment group; N vs. SCU: control group vs. the treatment group.

To understand the gene expression levels across samples, we calculated the expression values (TPM) for all genes in each sample. The distribution of gene expression levels among different samples is visualized via a density distribution plot, as shown in Figure 2A below. The horizontal axis represents log2(TPM + 1), while the vertical axis represents density.

#### 3.4.2. Sample Correlation Analysis

Principal Component Analysis (PCA) is also commonly used to assess intergroup differences and intra-group sample repeatability. Utilizing dimensionality reduction principles, PCA reduces the dimensionality of tens of thousands of gene variables and extracts principal components. To evaluate sample repeatability, we performed PCA on the gene expression values (TPM) of all samples. The results are shown in Figure 2B. In the PCA plot, samples from different groups are scattered, while samples within the same group cluster together, indicating good intra-group sample reproducibility.

#### 3.4.3. Differential Gene Analysis

Based on previous experiments, we know that scutellarin exhibits significant inhibitory activity against PRRSV at a concentration of 200 μg/mL. Therefore, for the transcriptomic analysis, we selected this concentration of scutellarin for experimentation and performed differential expression analysis using DESeq2.As shown in Figure 2C–F, Compared with the treatment group, the virus group exhibited 2644 differentially expressed genes, including 1011 genes that were up-regulated and 1633 genes that were down-regulated. And the control group showed 3217 differentially expressed genes, including 1354 up-regulated genes and 1863 down-regulated genes. Compared with the virus group, the control group showed 708 differentially expressed genes, including 448 up-regulated genes and 260 down-regulated genes. As shown in Figure 2G,H, 1955 genes were differentially expressed in both the control vs. treatment group and the challenge vs. treatment group comparisons. Additionally, 412 genes were differentially expressed in both the control vs. treatment group and the control vs. virus group comparisons.

#### 3.4.4. GO Enrichment Analysis of Differentially Expressed Genes

**Figure 3:**
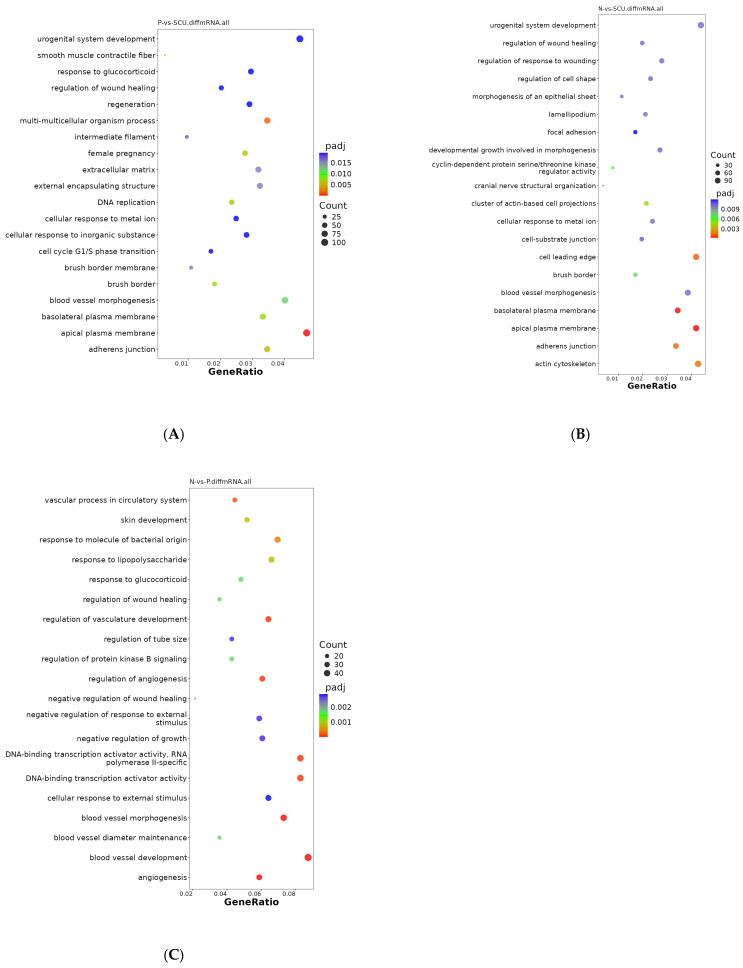
GO Enrichment Scatter Plots of Differentially Expressed Genes. (**A**) GO enrichment scatter plot of differentially expressed genes between the virus group and the treatment group. (**B**) GO enrichment scatter plot of differentially expressed genes between the control group and the treatment group. (**C**) GO enrichment scatter plot of differentially expressed genes between the control group and the virus group.

To elucidate the functions of differentially expressed genes, a GO analysis was conducted, encompassing molecular function (MF), cellular component (CC), and biological process (BP). From the GO enrichment analysis results, the top 20 terms were selected and visualized in a scatter plot (Figure 3).

As shown in Figure 3A, differentially expressed genes between the virus group and the treatment group were primarily distributed across cellular components and biological processes. Among these, differentially expressed genes associated with cellular components included: extracellular matrix, adherens junction, brush border, basolateral plasma membrane, smooth muscle contractile fiber, external encapsulating structure, intermediate filament, etc. Differentially expressed genes distributed across biological processes were primarily enriched in: multi-multicellular organism processes, DNA replication, female pregnancy, blood vessel morphogenesis, cellular response to metal ion, urogenital system development, cellular response to inorganic substance, regeneration, regulation of wound healing, and cell cycle G1/S phase. urogenital system development, cellular response to inorganic substance, regeneration, regulation of wound healing, cell cycle G1/S phase transition, and response to glucocorticoids.

Additionally, as shown in Figure 3B, differentially expressed genes between the control group and the treatment group were primarily enriched in CC-related terms, including: cyclin-dependent protein serine/threonine kinase regulator activity, cellular response to metal ion, focal adhesion, regulation of cell shape, developmental growth involved in morphogenesis, and others. Unlike the former, the differentially expressed genes between the control group and the virus group were primarily distributed in cellular components (Figure 3C), including: vascular processes in the circulatory system, DNA-binding transcription activator activity specific to RNA polymerase II, response to lipopolysaccharide, skin development, response to glucocorticoid, regulation of wound healing, regulation of protein kinase B signaling, negative regulation of wound healing, negative regulation of growth, negative regulation of response to external stimulus, cellular response to external stimulus, DNA-binding transcription activator activity, etc.

#### 3.4.5. KEGG Enrichment Analysis of Differentially Expressed Genes

**Figure 4:**
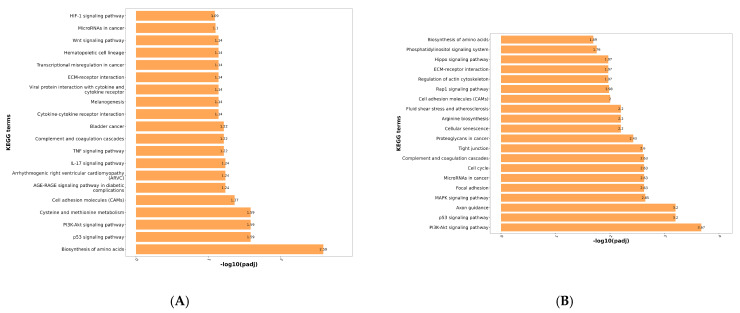

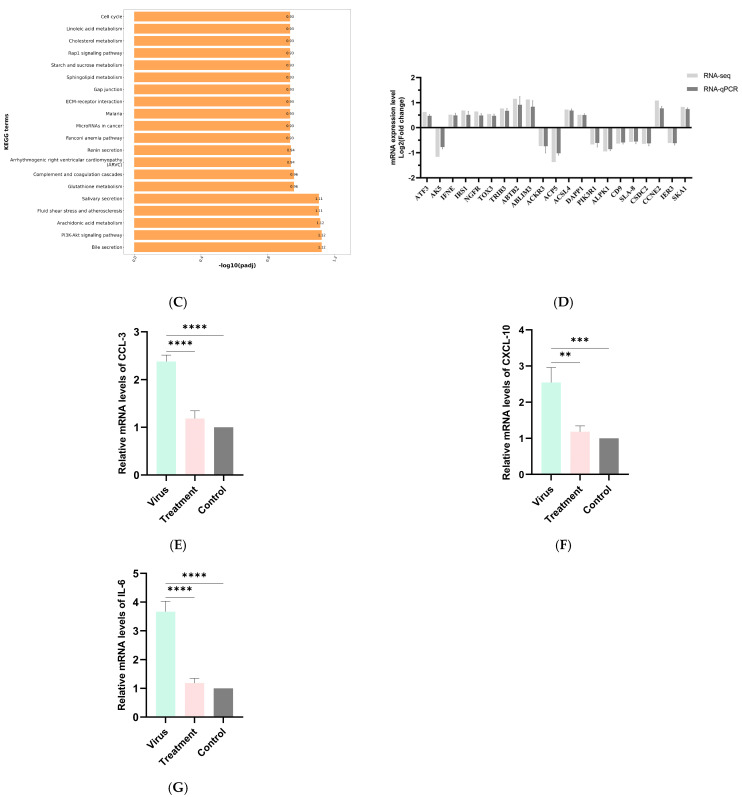
(**A**) KEGG enrichment bar chart of genes differing between control and virus groups. (**B**) KEGG enrichment bar chart of genes differing between control and treatment groups. (**C**) KEGG enrichment bar chart of genes differing between challenge and treatment groups. (**D**) qPCR validation results for the transcriptome. (**E**) The Results of qPCR on CCL-3 in PRRSV-Infected PAMs by scutellarin. (**F**) The Results of qPCR on CCL-3 in PRRSV-Infected PAMs by scutellarin. (**G**) The Results of qPCR on CCL-3 in PRRSV-Infected PAMs by scutellarin. *p* < 0.01, **; *p* < 0.001, ***, *p* < 0.0001, ****.

KEGG enrichment analysis was performed on the differentially expressed genes. From the enrichment results, the top 20 pathways (with the smallest padj values) were selected and visualized as a bar chart, as shown in Figure 4. The horizontal axis represents enrichment significance measured by −log10(padj), where higher values indicate greater significance. The vertical axis displays the KEGG pathways.

As shown in Figure 4A, differentially expressed genes between the control group and the virus group were primarily enriched in the following pathways: Biosynthesis of amino acids, PI3K-Akt signaling pathway, p53 signaling pathway, IL-17 signaling pathway, Complement and coagulation cascades, TNF signaling pathway, Cytokine-cytokine receptor interaction, ECM-receptor interaction, Viral protein interaction with cytokine and cytokine receptor, Wnt signaling pathway. Following PRRSV infection, the virus activates inflammation-related signaling pathways such as the PI3K-Akt signaling pathway, p53 signaling pathway, and cytokine-cytokine receptor interaction through its own encoded structural and non-structural proteins. This leads to the release of large amounts of cytokines and chemokines, inducing an inflammatory response. The occurrence of this inflammatory response can further influence PRRSV replication within the host [23,24].

As shown in Figure 4B, the differentially expressed genes between the control group and the treatment group were primarily enriched in the PI3K-Akt signaling pathway. Additionally, significant enrichment was observed in the MAPK signaling pathway, p53 signaling pathway, complement and coagulation cascades, tight junction, arginine biosynthesis, ECM-receptor interaction, Rap1 signaling pathway, and Hippo signaling pathway.

As shown in Figure 4C, differentially expressed genes between the virus group and the treatment group were primarily enriched in Bile secretion, PI3K-Akt signaling pathway, Arachidonic acid metabolism, Complement and coagulation cascades, Glutathione metabolism, Renin secretion, Cell cycle, Cholesterol metabolism, ECM-receptor interaction, Gap junction, Linoleic acid metabolism, Rap1 signaling pathway, and Sphingolipid metabolism. Inflammation, as one of the primary responses to PRRSV infection, plays a crucial role in the viral infection process. Viral replication and the production and interaction of numerous pro-inflammatory factors and chemokines drive the progression of PRRS, thereby exacerbating the inflammatory response. Based on KEGG enrichment analysis, we identified several inflammation-related signaling pathways, such as the PI3K-Akt signaling pathway and complement and coagulation cascades. This suggests that the mechanism of action of wild baicalin may be associated with responses related to inflammation regulation.

#### 3.4.6. qPCR Validation Results for Transcriptomes

To ensure the accuracy of transcriptomic results, qPCR was employed to validate sequencing data. Twenty-one genes (12 up-regulated and 9 down-regulated) were randomly selected from each group for validation. As shown in Figure 4D, the fold change (FC) values obtained from RT-qPCR generally aligned with the expression trends observed in transcriptomic sequencing results. This indicates that the transcriptomic data are reliable and suitable for subsequent data mining and analysis.

#### 3.4.7. The qPCR Results of Scutellarin on Inflammatory Factors in PRRSV-Infected PAMs

RT-qPCR was used to detect inflammatory factor levels in PAMs across all groups, with results shown in Figure 4E–G. Compared to the control group, PRRSV-infected PAMs in the virus group exhibited significant upregulation of CXCL10, CCL3, and IL-6. Compared to the treatment group, these inflammatory factors showed significant downregulation.

## 4. Discussion

As a major pathogen causing respiratory and reproductive diseases, PRRSV severely impacts the global swine industry. China continues to rely on vaccination to control PRRSV outbreaks, yet the use of PRRSV vaccines remains a highly controversial issue. On one hand, vaccines offer limited clinical protection and low efficacy; on the other, no better alternatives exist under current PRRS prevalence conditions. Major commercial vaccines in China include Ingelvac PRRS MLV, CH-1R, JXA1-R, HuN4-F112, and TJM-F92 [25,26,27]. While these commercial vaccines can partially control PRRSV outbreaks, alleviate clinical symptoms in pig herds, and reduce the duration of viremia, they cannot completely prevent PRRSV infection. Moreover, vaccines offer only partial or limited protection against heterologous challenges. Additionally, the misuse and overuse of vaccines have contributed to the emergence of new PRRSV strains, leading to greater strain complexity within pig populations. Zhang et al. [28] conducted a detailed genomic analysis of six PRRSV strains isolated from a single Chinese pig farm across different years. They found that all six strains resulted from recombination between wild-type strains such as HuN4-F112 and NADC30-Like and the MLV vaccine strain. This indicates that during vaccination with live attenuated vaccines, wild-type strains may recombine with vaccine strains to generate novel strains. It is precisely due to PRRSV’s inherent mutation potential and its capacity for recombination with vaccine strains and members of different lineages/sublineages that controlling PRRSV through vaccination has become exceptionally challenging. Furthermore, the recent ban on various antiviral drugs and the “antibiotic reduction trend” have further compounded the difficulty of PRRSV control. Therefore, there is an urgent need to explore a new anti-PRRSV drug.

In recent years, with the prohibition of antiviral drugs such as ribavirin, acyclovir, and guanidine in food animals, coupled with the current trend of “reducing and replacing antibiotics,” research on utilizing traditional Chinese medicine to treat and prevent PRRSV has increasingly gained favor among scholars. Sao et al. [29] found that Astragalus polysaccharides and white flower snake polysaccharides significantly increased the percentages of CD3+, CD4+, and CD8+ lymphocytes and specific antibody titers in piglets, thereby enhancing the immune response to the HP-PRRS inactivated vaccine. In vitro studies by Yu et al. [30] revealed that epigallocatechin gallate (EGCG), a polyphenolic compound in green tea, significantly mitigates PRRSV infection by inhibiting lipid synthesis and autophagy. Furthermore, studies indicate that various Chinese herbal medicines or their monomers—including Isatis root polysaccharides, Andrographis paniculata, Forsythia suspensa, and allicin—can significantly suppress PRRSV activity by promoting host immune function, inhibiting inflammatory responses, and blocking viral replication [31,32,33]. These findings collectively demonstrate the immense potential of traditional Chinese medicine in preventing and controlling PRRSV.scutellarin, a natural flavonoid compound extracted from traditional Chinese medicinal herbs such as *Scutellaria baicalensis*, *Scutellaria barbata*, and *Erigeron chrysanthemoides*, exhibits multiple biological functions including antiviral, antioxidant, immune-enhancing, antitumor, and anti-inflammatory effects. Current research on its biological functions primarily focuses on antitumor and anti-inflammatory effects, with significant gaps remaining in antiviral studies. Only limited research indicates that scutellarin can inhibit viral activity by suppressing the fusion of viral envelopes with cell membranes and inhibiting viral helicase [20,21,22]. While it demonstrates inhibitory effects against viruses such as SARS-CoV and HIV, no reports currently exist regarding its antiviral activity against PRRSV. Therefore, this study employs in vitro cell assays and transcriptomic analysis to investigate the inhibitory effect of wild scutellarin on PRRSV-infected PAMs. Based on transcriptomic screening, it identifies differentially expressed genes involved in this mechanism, aiming to provide a research foundation and scientific basis for subsequent studies on its mechanism and antiviral drug development against PRRSV.

In this study, we determined the maximum safe concentration of scutellarin for PAMs to be 200 μg/mL using the CCK-8 assay. At this concentration, we conducted in vitro cell experiments with gradient dilutions. We found that at 200 μg/mL, scutellarin exhibits both preventive and inhibitory effects against PRRSV, with its inhibitory effect being more pronounced. Focusing on its inhibitory effect, we performed sample preparation and transcriptome sequencing. Comprehensive analysis of differential gene expression clustering and functional enrichment results provided a more detailed understanding of the molecular basis underlying scutellarin’s in vitro inhibition of PRRSV. GO enrichment analysis revealed that differentially expressed genes function in cellular structure, metabolic processes, and immune responses.

KEGG enrichment analysis revealed that compared to the control group, differentially expressed genes in the virus group were primarily enriched in immune and inflammation-related signaling pathways. These included the PI3K-Akt signaling pathway, p53 signaling pathway, IL-17 signaling pathway, complement and coagulation cascades, TNF signaling pathway, cytokine-cytokine receptor interaction, ECM-receptor interaction, and viral protein interaction with cytokine and cytokine receptor. The activation of these pathways reflects that PRRSV infection induces a robust immune response. Notably, the expression of pathways such as Complement and coagulation cascades, ECM-receptor interaction, Rap1 signaling pathway, and PI3K-Akt signaling pathway in both the experimental and virus groups may involve innate immune and anti-inflammatory mechanisms. Additionally, RT-PCR analysis revealed that CXCL10, CCL3, and IL-6 were significantly upregulated in PRRSV-infected PAMs from the virus group compared to the control group. However, these inflammatory factors were markedly downregulated in the treatment group. Thus, it is reasonable to infer that scutellarin may influence PRRSV-infected PAMs by modulating the inflammatory response.

In summary, through in vitro experiments and transcriptomic analysis, we hypothesize that scutellarin may influence PRRSV infection of PAMs by regulating host inflammatory responses, thereby affecting host metabolism and function. This provides a direction and research foundation for our subsequent investigation into its specific mechanisms.

## Figures and Tables

**Table 1:** Maximum Safe Concentration of scutellarin for PAM Cells.

Concentration (μg/mL)	OD450 (Mean ± SD)	Suppression Rate (%)
800	0.7868 ± 0.005	31.99
600	0.8725 ± 0.006	22.20
400	0.9133 ± 0.013	17.55
200	1.220 ± 0.016	5.14
100	1.0399 ± 0.012	3.10
50	1.0679 ± 0.015	−0.10
Cell Control	1.0672 ± 0.070	0.00
Blank Control	0.1915 ± 0.007	100.00

## Data Availability

The raw data supporting the conclusions of this article will be made available by the authors on request.

## Appendix A

**Table A1:** Primers used for PCR validation in this study.

Primer Name	Primer Sequence (5′-3′)
pcDNA3.1-M-F	GCTGGCTAGCGTTTAAACTTAAGCTTGCCACCATGGGGTCGTCTCTAGACG
pcDNA3.1-M-R	CACTGTGCTGGATATCTGCAGAATTCTTACTTGTCATCGTCGTCCTTGTAATCTTTGGCATATTTGACAAGGTTTACC
β-actin-F	CTCCATCATGAAGTGCGACGT
β-actin-R	GTGATCTCCTTCTGCATCCTGT
PRRSV-qF	TTGCTAGGCCGCAAGTAC
PRRSV-qM	ACGCCGGACGACAAATGC
PRRSV-P	CTGGCCCCTGCCCACCAC
ATF3-F	TGGCGGCGGCAATCTTAT
ATF3-R	TGGCGGCGGCAATCTTAT
AK5-F	GCCAATCTCAGCAACACCAA
AK5-R	GCCGAGCAGTCCATACAGA
IFNE-F	TGTTGGTACTGCTGGCTTCT
IFNE-R	ACTGCTGAATTGATGAGGTCTG
IRS1-F	GACCAGCAAGACCATCAG
IRS1-R	GCCACCACAGAATCATCC
NGFR-F	CCTGTCTATTGCTCCATCC
NGFR-R	TTGGCTCCTTGCTTGTTC
TOX3-F	ACCACCATCAACCAGTCT
TOX3-R	ATTCATCAGCGTCCTCTTC
TRIB3-F	GAAGAAGCGGTTGGAGTT
TRIB3-R	CTCAGAAGCAGTGGACAG
ABTB2-F	CCATCAAGGAGGAGGAATAC
ABTB2-R	CACGAGGAAGGTCACATC
ABLIM3-F	CTCGCCTCACCACTACTA
ABLIM3-R	AACTCATCCTCCTCTCCTC
ACKR3-F	TTCGGCAGCATCTTCTTC
ACKR3-R	AGTAGGTCTCGTTGTTGGA
ACP5-F	CAGGATGAGAATGGCTTGG
ACP5-R	CCTTGGCAACTTGGTCTT
ACSL4-F	ATGTCCGTATGATGCTGTC
ACSL4-R	GGCTTGTCGTGAACTGTA
DAPP1-F	AGGCACTCTCATTGTTCTG
DAPP1-R	AGGTCGTTCTCTGTTCTTC
PIK3R1-F	TGGAATGTCGGAAGCAGCAACC
PIK3R1-R	ACAGAGCAGGCGTAGCAACC
ALPK1-F	CCTATTCACAGCACCACACTTC
ALPK1-R	CTCCAGACCACTCTTAGCCATT
CD9-F	ATTGCGGTCCTTGCCATTG
CD9-R	GGAAGCCCACCACCATCAT
SLA-8-F	GTTGCTGGTCTGGTTCTCCT
SLA-8-R	CGACGCTGTTGTTGCTTGG
CSDC2-F	GCAGTTCTCACGCTCACAG
CSDC2-R	GTCACCTCATCGCCTTCCA
CCNE2-F	GCGTATGTCACTGATGGT
CCNE2-R	GAGAATACTGAGGTAGAAGAAC
IER3-F	CTGGTCCCGAGATATTCAC
IER3-R	ACAGAAGACGATGGTAAGC
SKA1-F	GTGATGTTCGTTGCTGTC
SKA1-R	GCCGTGATGTAGAGAAGG
CCL3-F	CTGGTGAGGAACGACAAG
CCL3-R	ACATAGGCTTGAGGTCATAC
CXCL10-F	CTGAGAGTGATTGAGAGTGGAC
CXCL10-R	TACTGCTGTTGTTGTTGCTTCT
IL6-F	CCTTCAGTCCAGTCGCCTTCTC
IL6-R	GCATCACCTTTGGCATCTTCTTCC
